# Impact of Small Intestine Bacterial Overgrowth on Response to a Nutritional Intervention in Bangladeshi Children from an Urban Community

**DOI:** 10.4269/ajtmh.18-0759

**Published:** 2018-11-26

**Authors:** S. M. Abdul Gaffar, Shafiqul Alam Sarker, Mustafa Mahfuz, Jeffrey R. Donowitz, Tahmeed Ahmed

**Affiliations:** 1 Nutrition and Clinical Services Division (NCSD), International Centre for Diarrhoeal Disease Research, Bangladesh (icddr,b), Dhaka, Bangladesh;; 2 Division of Pediatric Infectious Diseases, Children’s Hospital of Richmond at Virginia Commonwealth University, Richmond, Virginia

## Abstract

Small intestine bacterial overgrowth (SIBO) is prevalent among children living in low-income settings, leading to impaired growth and development. The aim of this study was to assess linear and ponderal growth parameters between malnourished SIBO-positive and SIBO-negative children aged 12–18 months who prospectively underwent a nutritional intervention. A glucose hydrogen breath test to detect SIBO was performed in 194 stunted (length-for-age *Z* score [LAZ] < −2 standard deviations) or at-risk of stunting (LAZ score between < −1 and −2 standard deviations) children. Participants received nutritional supplementation (egg and milk) in addition to their regular family meals 6 days per week for 90 days. Small intestine bacterial overgrowth was defined as a ≥ 12-ppm rise in breath hydrogen over the patient’s baseline during the 3-hour test. Small intestine bacterial overgrowth status before intervention was forced into a multivariable linear regression model to examine its effects on anthropometric changes in response to the intervention. Sociodemographic data at enrollment was analyzed through multivariable logistic regression in an attempt to predict SIBO positivity. Overall, 14.9% (29/194) children were diagnosed with SIBO before the nutritional intervention. No statistically significant difference was observed among SIBO-positive and SIBO-negative groups in terms of their response to the nutritional intervention (SIBO-positive coefficient [95% confidence interval (CI)], *P*-value for ∆length-for-age Z score −0.003 [−0.14, 0.13], 0.96; ∆weight-for-age *Z* score −0.05 [−0.20, 0.09], 0.46; and ∆weight-for-length *Z* score −0.10 [−0.31, 0.10], 0.33). This study demonstrated that a noteworthy proportion of malnourished children living in a disadvantaged urban community were SIBO positive; however, it failed to reveal an association between SIBO status and response to nutritional intervention.

## INTRODUCTION

Small intestine bacterial overgrowth (SIBO) represents an increased number (≥ 10^5^ colony-forming unit [CFU]/mL) of bacteria present in the upper small intestine.^1^ Several studies have shown that a significant number of children from low- and middle-income countries (LMICs) living in impoverished conditions were SIBO positive as diagnosed by hydrogen breath tests.^2,3^ A study of children aged 6–10 years in Brazil found that 30.9% of lower socioeconomic status children were SIBO positive compared with 2.4% children with increased financial means.^4^ Small intestine bacterial overgrowth has also been associated with environmental enteropathy and intestinal inflammation.^5^ The prevalence of SIBO among 2-year-old Bangladeshi children was 16.7% based on a recent cross-sectional study which demonstrated an association between linear growth faltering and glucose hydrogen breath test positivity.^5^

The consequences of SIBO and its long-term implications on child health are still unclear. No study comparing the effect of SIBO on nutritional interventions has been reported. Moreover, the risk factors for the development of SIBO in children living in LMICs remain largely unexplored. Several studies have shown an association between SIBO positivity and lower socioeconomic status and one study demonstrated markers of fecal–oral contamination predicted the presence of SIBO.^2,4,5^

The aim of our study was to examine the growth differences between SIBO-positive and SIBO-negative children from an impoverished urban area who completed a community-based nutrition intervention program. In addition, we wanted to evaluate demographic and socioeconomic factors which might predict the development of SIBO.

## METHODS

Participants for this study were selected from the ongoing Bangladesh Environmental Enteric Dysfunction (BEED) study. The socioeconomic and demographic characteristics of the study area have been described elsewhere.^6,7^ Briefly, the BEED study involves a community-based nutritional intervention program in children aged 12–18 months who were stunted (length-for-age *Z* score [LAZ] < −2) or at risk of stunting (LAZ < −1 to −2). A subset of children who fail nutritional therapy will go on to have upper gastrointestinal endoscopy. The primary objective of the BEED study is to develop a histological scoring system for the diagnosis of environmental enteric dysfunction. Enrolled children received dietary supplementation with one boiled egg and 150 mL of whole milk daily for 90 days, which provided an additional 178 kilocalories, 11.1 g of protein, and 11.5 g of fat daily on top of their normal home meals. In addition, one sachet of micronutrient sprinkles comprising vitamins A and C, folic acid, iron, and zinc was added to the supplements for 60 days. Supplementation was given via directly observed therapy at nutrition centers established in the community. The BEED study was approved by the Research and Ethical Review Committees of the International Centre for Diarrhoeal Disease Research, Bangladesh (icddr,b).

All children had a glucose hydrogen breath test (GHBT) performed before starting the nutritional intervention. Children with antibiotic use in the 14 days before scheduled GHBT were rescheduled, ensuring a 14-day antibiotic-free period before testing. Glucose solution was administered orally at a dose of 1 g/kg body weight dissolved in 5 mL/kg water after a baseline breath sample was collected. Breath samples were collected every 20 minutes thereafter for 3 hours using the Quintron infant/child breath collection system. Briefly, this system involves an age-appropriate anesthesia mask connected to a collection bag via a one-way flutter valve. Samples were pulled from the bag using a syringe and stopcock as per the manufacturer’s recommendation and immediately run on a QuinTron BreathTracker SC gas chromatograph (QuinTron Instrument Company, Inc., Milwaukee, WI). Small intestine bacterial overgrowth positivity was defined as a value of breath hydrogen ≥ 12 ppm over the baseline measurement on any single post-glucose reading. The GHBT was repeated after completion of the nutritional intervention in those participants who were SIBO-positive before the intervention.

Weights and lengths of the participants were measured before and after the nutritional intervention using standard scales (Seca GmbH & Co. KG., Hamburg, Germany). Data on maternal age, education, and handwashing practice before food preparation as well as source of drinking water, number of people sleeping per room, and average monthly income of the entire household were collected using a questionnaire at the time of enrollment. Maternal age was discretized to ≥ 30, 20–29, and ≤ 19 years. Maternal education was discretized into no formal education, primary education only, and secondary education or above. Handwashing practice was discretized to never, rarely, sometimes, and always. Source of drinking water was dichotomized to pumped into the home versus other. People sleeping per room was dichotomized to ≥ 3 and < 3. Average monthly household income was converted from Bangladeshi taka to USD (82.96 BDT = 1 USD) and analyzed as a continuous variable.

Predictors of SIBO positivity at enrollment were explored using multivariable logistic regression. The objective of this analysis was to identify sociodemographic predictors of SIBO positivity. We selected nine covariates for logistic regression to predict the presence of SIBO at enrollment. Each covariate was first analyzed for an association with SIBO status by univariate regression. Variables significant at the 0.25 level were included in the multivariable analysis.

Response to nutritional therapy of SIBO-positive and SIBO-negative subjects was compared using multivariable linear regression. The objective was to build a comprehensive model to examine the effect of SIBO on our outcome variables (∆length-for-age Z score [LAZ], ∆weight-for-age *Z* score [WAZ], and ∆weight-for-length *Z* score [WLZ]). Covariates included the breath test outcome (SIBO positive or negative), gender (male or female), and pre-intervention age. Outcome variables were derived by subtracting pre-intervention from post-intervention LAZ, WAZ, and WLZ. Covariates that were significant at the 0.25 level by univariate regression analysis were included in the final multivariable model. This process was repeated for the three separate regressions for each of our three outcome variables (∆LAZ, ∆WAZ, and ∆WLZ). Breath test outcome was forced into the multivariable models. All statistical analyses were computed using STATA version 13.0 (StataCorp LP, College Station, TX).

## RESULTS

One hundred ninety-four participants who completed the nutritional intervention and underwent pre-intervention GHBT were included in our analysis. Of them, 92 were stunted and 102 were at risk of stunting. The demographic characteristics of the participants before and after the nutritional intervention are presented in Table 1. The overall prevalence of SIBO was 14.9% (29/194). It was 16.3% (15/92) in the stunted group and 13.7% (14/102) in the at-risk-of-stunting group. The incidence of SIBO was not significantly different in females (19.3%; 21/109) versus males (9.4%; 8/85) (*P*-value 0.07). Of the 29 SIBO-positive participants, 20 had repeat GHBT after the nutritional intervention. The nine others had parents who refused retesting. In all, only four of the 20 remained SIBO positive on repeat testing.

**Table 1:** Anthropometric characteristics of participants at pre- and post-nutritional intervention

Parameter	Mean (±standard deviation)	
	Pre-intervention (*n* = 194)	Post-intervention (*n* = 194)
Age (months)	14.72 ± 2.18	18.35 ± 2.18
Length-for-age *Z* score	−2.16 ± 0.79	−2.08 ± 0.79
Weight-for-age *Z* score	−1.82 ± 0.85	−1.84 ± 0.85
Weight-for-length *Z* score	−1.04 ± 0.87	−1.14 ± 0.86

For our primary outcome of the effect of SIBO on response to the nutritional intervention, we did not observe any statistically significant effect of SIBO on growth parameters in response to the nutritional intervention in the univariate regression analysis. Small intestine bacterial overgrowth remained nonsignificant when forced into our model. The details of the multivariable linear regression analysis are listed in Table 2.

**Table 2:** Relationship between glucose hydrogen breath test and anthropometric attainment of children who underwent nutritional intervention

Variables		Unadjusted coefficient* (95% CI)	*P*-value	Adjusted coefficient† (95% CI)	*P*-value
∆LAZ	SIBO (+)	−0.008 (−0.14, 0.12)	0.90	−0.003 (−0.14, 0.13)	0.96
	Age	0.02 (−0.001, 0.04)	0.07	0.02 (−0.001, 0.04)	0.07
∆WAZ	SIBO (+)	−0.07 (−0.22, 0.08)	0.35	−0.05 (−0.20, 0.09)	0.46
	Female	−0.08 (−0.18, 0.02)	0.14	−0.07 (−0.18, 0.03)	0.17
∆WLZ	SIBO (+)	−0.11 (−0.32, 0.09)	0.29	−0.10 (−0.31, 0.10)	0.33
	Age	0.03 (0.002, 0.07)	0.04	0.03 (0.001, 0.07)	0.04

∆LAZ, ∆WAZ, and ∆WLZ denote change in length-for-age, weight-for-age, and weight-for-length *Z* scores during the intervention period; SIBO (+) denotes small intestine bacterial overgrowth positive; Age indicates pre-intervention age in months. For each outcome, only covariates that were significant at *P* ≤ 0.25 were included.

* Univariate linear regression.

† Multivariable linear regression.

For our secondary analysis to predict SIBO from sociodemographic variables, we excluded group (*P* = 0.61 for stunted; reference at-risk-of-stunting children), pre-intervention age (*P* = 0.57) of the participants, education of the mothers (*P* = 0.65 for primary and *P* = 0.78 for secondary and higher; reference no formal education), and income of the household (*P* = 0.72) from our multivariable model based on univariate *P*-values > 0.25. However, gender of the participants (*P* = 0.06 for female; reference male), maternal age (*P* = 0.17 for ≤ 19 years; reference ≥ 30 years), handwashing practice before preparation of food of the mothers (*P* = 0.14 for sometimes; reference never), source of drinking water (*P* = 0.16 for other than piped into dwelling; reference piped into dwelling), and number of people sleeping per room (*P* = 0.04 for < 3; reference ≥ 3) were included in the final multivariable analysis.

In our multivariable model, none of the covariates achieved statistical significance at the < 0.05 level for the prediction of SIBO. The details of the multivariable logistic regression are listed in Table 3.

**Table 3:** Predictors of small intestine bacterial overgrowth positivity at enrollment (*n* = 194)

Variables	Unadjusted OR (95% CI)	*P*-value	Adjusted OR (95% CI)	*P*-value
Gender of the participant				
Male	Reference		Reference	
Female	2.30 (0.96, 5.48)	0.06	2.28 (0.92, 5.62)	0.07
Age of the mother (years)				
≥ 30	Reference		Reference	
≤ 19	2.62 (0.67, 10.30)	0.17	1.75 (0.39, 7.83)	0.46
20–29	1.49 (0.52, 4.25)	0.45	1.16 (0.36, 3.69)	0.80
Drinking water quality				
Piped into dwelling	Reference		Reference	
Others	0.57 (0.26, 1.26)	0.16	0.60 (0.25, 1.44)	0.26
Mother’s hygiene (wash hands with soap before preparing food)				
Never	Reference		Reference	
Rarely	0.75 (0.15, 3.67)	0.72	0.72 (0.14, 3.68)	0.69
Sometimes	0.47 (0.17, 1.29)	0.14	0.39 (0.13, 1.13)	0.08
Always	1.72 (0.58, 5.08)	0.33	1.21 (0.38, 3.87)	0.75
People sleeping in the household per room				
≥ 3	Reference		Reference	
< 3	2.34 (1.02, 5.33)	0.04	2.08 (0.81, 5.39)	0.13

OR = odds radio.

## DISCUSSION

The purpose of this study was to evaluate SIBO’s effect on children’s response to a community-based nutritional intervention. We found no difference in growth parameters between SIBO-positive and SIBO-negative participants in response to our intervention. A recent cross-sectional analysis of Bangladeshi children had a similar rate of SIBO to that of our cohort^5^ found an association between SIBO positivity and preceding linear growth delay. The reason we did not see the expected impact of SIBO on the efficacy of our nutritional intervention may be because of several factors. First, the cohorts comprised of different populations. We enrolled only stunted and at-risk-of-stunting children, whereas the previous analysis used a population that included stunted and non-stunted children. Moreover, the previous analysis used a cohort that was observed longitudinally for 2 years, whereas we estimated growth for a period of three and half months. It may be that SIBO’s effect size on growth is too small to have been detected in our short timeline. Several studies have described the various harmful impact of SIBO on nutrient absorption in high-income settings. Poor absorption of thiamine, vitamin B_12_, and the fat-soluble vitamins (excluding vitamin K) in adult patients with other comorbid conditions has been described.^8–12^ Only a limited number of studies have been conducted among the pediatric population living in unsanitary conditions in low-income countries to examine the impact of SIBO on nutritional parameters.^13–15^ In Burmese studies, SIBO-positive children aged 1–59 months were found to be carbohydrate malabsorbers, which was significantly associated with growth faltering.^13,15^ A Nigerian study found that malnourished children with or without diarrhea had a higher prevalence of SIBO in contrast to their well-nourished counterparts.^14^ Again, the reason we did not see an association between nutritional outcomes and SIBO status may have been the short timeline of our study.

Our study findings suggest that SIBO had no impact on response to nutritional therapy among children aged less than 2 years who were either stunted or at risk of stunting. Moreover, several sociodemographic factors failed to predict the occurrence of SIBO in our study population. However, it is evident from our study that a considerable number of children were suffering from SIBO, the exact cause of which is yet to be explored. Previous studies conducted in the developing countries also revealed higher prevalence of SIBO among the pediatric population. A Burmese study of 340 village children aged 1–59 months found a 27.2% prevalence of SIBO (53/195) with a significant male predominance.^3^ The same study found that diarrhea had no impact on breath H_2_ excretion among the H_2_ producers. Another study conducted in Brazil which included 50 asymptomatic underprivileged children aged 5–11 years and 50 age- and gender-matched controls selected from a private clinic found a significantly greater prevalence of SIBO among underprivileged children (37.5% versus 2.1%, *P*-value < 0.001).^2^

Intestinal dysbiosis may occur among the children, particularly who are suffering from chronic malnutrition due to the inadequate dietary intake of essential nutrients.^16^ Theoretically, dysbiosis may lead to overgrowth of a pathologic bacterial population, which ultimately produces inflammation in the gut.^16^ Several studies have evaluated the impact of diet on the human gut microbiota. One study revealed that rural African children consuming a high-fiber diet were protected against pathogenic bacteria in comparison to European children who were on a high-fat diet.^17^ In another study, a Mediterranean-inspired anti-inflammatory diet composed of fruits, vegetables, grains, and monounsaturated or n-3 polyunsaturated fats was found to reduce inflammation in Crohn’s disease and normalize the gut microbiota.^18^ This may be an explanation of why many of the SIBO-positive children became negative during the course of the study. However, as we do not know the natural history of this condition in the setting of environmentally derived SIBO in low-income countries, it may be that the observation was coincidental. The need for a future study including a control group is indicated.

The unique strength of our study was its prospective design to evaluate the response to a nutritional therapy of SIBO-positive and SIBO-negative participants for a certain period of time. Using directly observed therapy ensured a true intervention.

There are several important limitations of our study. First was the relatively short period of time that we followed up our participants. A longer intervention may have allowed us to detect a small effect size of SIBO on the nutritional intervention. Second, we lacked a control group who did not receive the nutritional intervention. This would have allowed us to ascertain the impact of the intervention on SIBO status. In addition, our dichotomous cutoff of ≥ 12 ppm is based on adult studies and may not be optimal for our target population, given that it is based on adult studies in high-income populations.^19^

## CONCLUSION

Small intestine bacterial overgrowth was common among the children in our study population, stunted or at risk of stunting in a low-income setting. Further research is needed to elucidate the current risk factors in children living in low-income countries which contribute to the development of SIBO in early childhood and to determine if there is a negative impact on growth and development in longer duration longitudinal studies.
